# A case report of classic galactosemia with a *GALT* gene variant and a literature review

**DOI:** 10.1186/s12887-024-04769-0

**Published:** 2024-05-22

**Authors:** Yong-cai Wang, Lian-cheng Lan, Xia Yang, Juan Xiao, Hai-xin Liu, Qing-wen Shan

**Affiliations:** 1 Difficult and Critical Illness Center, Pediatric Clinical Medical Research Center of Guangxi, The First Affiliated Hospital of Guangxi Medical University, No. 6 Shuangyong Road, Nanning, 530021 Guangxi Zhuang Autonomous Region China; 2 Dazhou Central Hosptial, No. 56 Nanyuemiao Street, Tongchuan District, Dazhou, 635000 Sichuan Province China

**Keywords:** Galactosemia, *GALT* gene, Exome sequencing, Liver failure, Literature review

## Abstract

**Background:**

Galactosemia is an autosomal recessive disorder resulting from an enzyme defect in the galactose metabolic pathway. The most severe manifestation of classic galactosemia is caused by galactose-1-phosphate uridylyltransferase (*GALT*) deficiency, and this condition can be fatal during infancy if left untreated. It also may result in long-term complications in affected individuals.

**Case presentation:**

This report describes a patient whose initial clinical symptoms were jaundice and liver dysfunction. The patient’s liver and coagulation functions did not improve after multiple admissions and treatment with antibiotics, hepatoprotective and choleretic agents and blood transfusion. Genetic analysis revealed the presence of two variants in the *GALT* gene in the compound heterozygous state: c.377 + 2dup and c.368G > C (p.Arg123Pro). Currently, the variant locus (c.377 + 2dup) in the *GALT* gene has not been reported in the Human Gene Mutation Database (HGMD), while c.368G > C (p.Arg123Pro) has not been reported in the Genome Aggregation Database (GnomAD) nor the HGMD in East Asian population. We postulated that the two variants may contribute to the development of classical galactosemia.

**Conclusions:**

Applications of whole-exome sequencing to detect the two variants can improve the detection and early diagnosis of classical galactosemia and, more specifically, may identify individuals who are compound heterozygous with variants in the *GALT* gene. Variants in the *GALT* gene have a potential therapeutic significance for classical galactosemia.

## Introduction

Galactosemia is an autosomal recessive disorder resulting in galactose accumulation in the body due to defective enzyme function-associated disorders of the galactose metabolic pathway, which interfere with normal cell physiological functions [1]. Classical galactosemia (also known as type I galactosemia) is caused by the deficiency of galactose-1-phosphate uridylyltransferase (*GALT*). The *GALT* gene is located on chromosome 9p13, has a length of approximately 4.3 kb, and consists of 11 exons. The mRNA sequence is about 1.2 kb long and encodes 379 amino acids. The *GALT* protein is a homodimer with two identical functional sites, including a His-Pro-His motif [2]. It is the most severe form among the three forms. Classic galactosemia symptoms typically appear in early life after consuming breast milk and/or formula containing lactose for a few days. The early symptoms include feeding difficulties, vomiting, hypoglycemia, diarrhea, jaundice, hepatomegaly, liver dysfunctions, lethargy, hypotonia, low eye pressure, renal tubular disease, cataracts, and sepsis [3, 4].

Untreated classical galactosemia often leads to fatal outcomes during infancy. Therefore, early cessation of lactose-containing (galactose-containing) foods can alleviate or eliminate the complications. However, some children may develop long-term complications when *GALT* enzyme activity are deficient or severely reduced. Galactosemia-affected children have been reported with brain injuries (85%), and decreased bone densities (26.5%) [5]. Therefore, early detection and treatment with a lactose-restricted diet is recommended. The dietary treatment results in a gradual resolution of the child’s jaundice and ascites and leads to a significant improvement in coagulation and liver function.

Clinical variant galactosemia, commonly seen in African American individuals, is homozygous for the c.404 C > T (p.S135L) variant and *GALT* activity are 8–12% in the liver and intestinal epithelial cells [3, 6, 7]. Untreated neonatal hypergalactosemia with Gal-1-P levels > 10 mg/dL can result in acute symptoms in the newborn period, including growth restriction, liver disease, and cataracts. A lactose-restricted diet can help prevent the diet-independent chronic complications associated with classic galactosemia. If galactosemia is suspected, even based on clinical findings alone, lactose in the diet should be immediately restricted.

**Fig. 1:**
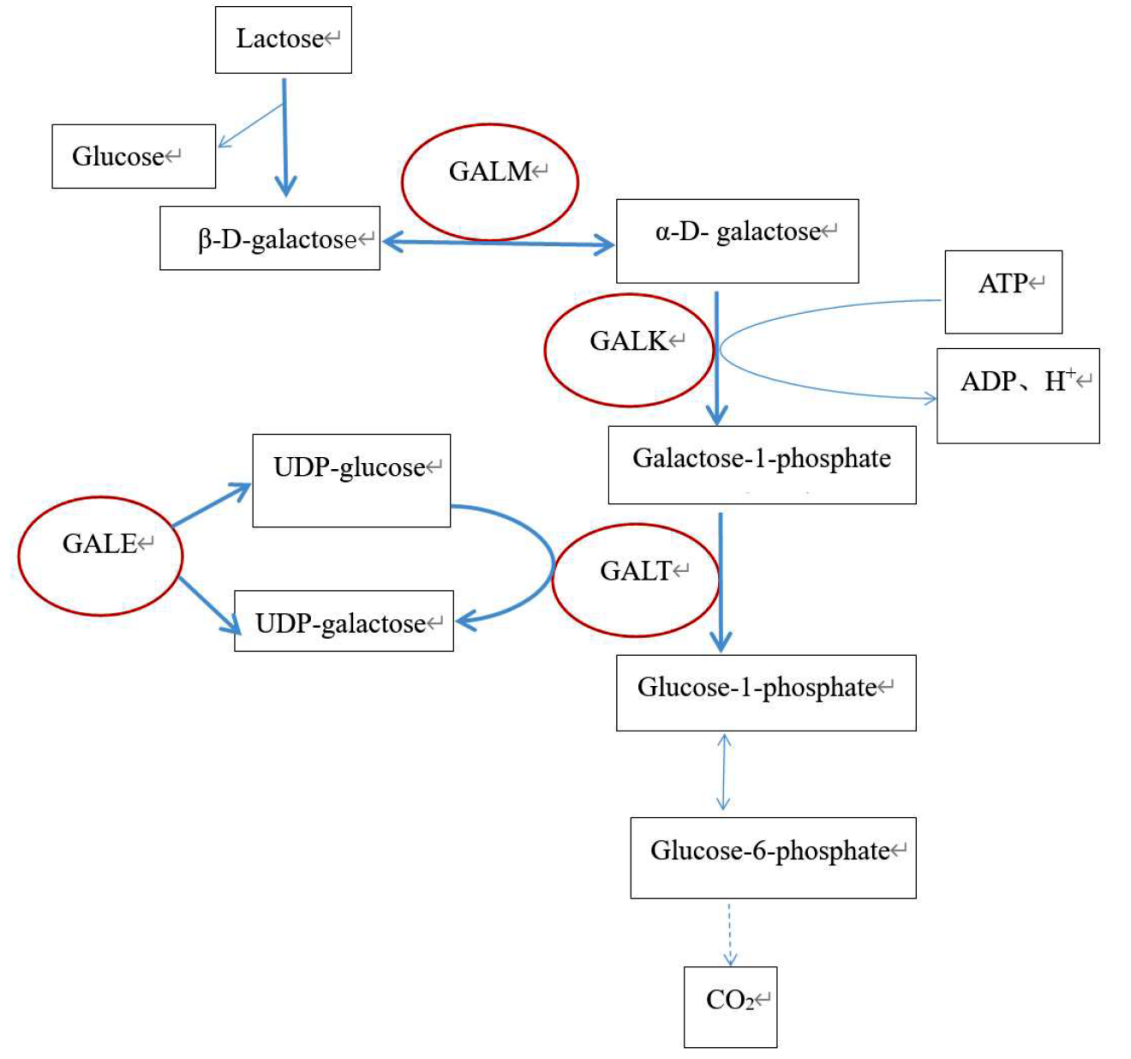
Represents the standard Leloir pathway

Lactose in breast milk and dairy products is hydrolyzed into galactose and glucose in the presence of intestinal lactase, after which galactose is absorbed into the blood via the intestines and metabolized in the liver via the Leloir pathway (Fig. 1). This pathway consists of four steps, each catalyzed by a different enzyme. Galactose epimerase (GALM) promotes the conversion of β-D-galactose to ɑ-D-galactose, while the subsequent step involves the conversion of ɑ-D-Galactose to galactose-1-phosphate (Gal-1-P) by galactokinase (GALK). In the third step, *GALT* converts UDP-glucose (UDP-Glu) and Gal-1-P to glucose-1-phosphate (GLU-1-P) and UDP-galactose (UDP-Gal). The final step is driven by the UDP-galactose-4´-epimerase (GALE) enzyme, which catalyzes the conversion of UDP-Gal to UDP-Glu [8, 9]. Ultimately, Glu-1-P enters the glycolytic pathway.

The alternative pathway of galactose metabolism is particularly active when there are deficiencies in Leloir pathway-associated enzymes, which can lead to the accumulation of galactose and other abnormal metabolites in the body, affecting the liver, kidneys, eyes, and brain and can be fatal. Due to the toxic effects of galactose metabolites, survivors may develop various long-term complications, such as cataracts, abnormal neurological development, speech impairment, growth restriction, and premature ovarian failure [10, 11].

Blood tandem mass spectrometry screening for galactosemia is not routinely performed in China, while conventional laboratory tests make it challenging to diagnose the disease. In many countries, including those following international guidelines, the enzymatic assay, one of the recommended techniques, is no longer commonly performed and has been largely replaced by the sequencing of the *GALT* gene. However, in China, genetic sequencing is the primary method for diagnosing certain conditions, but it is often outsourced to third-party institutions, resulting in a long wait time for results. Galactosemia is a rare condition, and clinicians may not always be aware of it, which can result in misdiagnosis or missed diagnosis, as was the case presented in this report. There are no specific drugs for galactosemia treatment. Immediate dietary galactose restriction is required to reverse the acute post-natal symptoms of classic galactosemia.

The prevalence of classical galactosemia among populations of different ethnic backgrounds varies. Its prevalence is much higher in Western populations compared to Asian populations, with prevalence rates of 1:40,000∼1:60,000 in Europe, 1:50,000 in the United States, 1:23,500–1:44,000 in the United Kingdom, 1:42,000 in Lithuania, 1:100,000 in Japan, 1:400,000 in Taiwan, China [12], 1:50,000 in Shenzhen, China 1:50,000, and 1:759,428 in Zhejiang, China 1:759,428 [13]. The disease is less reported in China. The new case of the *GALT* gene variant reported in this study will expand its phenotype and genetic profile.

## Study methodology

### Ethical approval

The Ethical Review Committee of The First Affiliated Hospital of Guangxi Medical University, China, approved this study (Approval Number: 2023-E183-01). All examinations and experiments were conducted following the Declaration of Helsinki. The patient’s mother and father agreed to genetic analysis and signed informed consent.

### Case presentation

#### Patient information, personal and family history

The patient was born at term with no complications and was the product of the fourth pregnancy, 3rd delivery to non-related parents. The patient was initially breastfed. On the 3rd day after birth, she exhibited yellowing of the skin on the face, which did not improve after phototherapy. She was referred to the local maternal and child health hospital for her first hospitalization. Abnormal liver and coagulation functions were found during clinical examination (Tables 1 and 2). Urine tests for organic acid revealed elevated levels of 4-hydroxyphenyl, adipic, suberic, and sebacic acids, while the amino acids, acylcarnitine, and succinylacetone levels in blood normal. The patient was put on a two-day fasting regimen, administered with antibiotics and Hepatoprotective (compound glycyrrhizin injection 8 ml added to 50 ml glucose (5%) intravenous infusion, once a day) and choleretic treatments (ursodeoxycholic acid capsules 25 mg orally once daily). She was transfused with plasma and vitamin K1. Total bilirubin levels decreased to 87.2 umol/L (direct bilirubin: 60.6 umol/L) and the skin improved. After discharge from the hospital, her complexion gradually became pale, and reexamination revealed abnormal liver functions and moderate anemia.

The patient was readmitted to the local maternal and child health hospital (second hospitalization) for further treatment. Reexamination revealed continued abnormal liver and coagulation functions (Table 1), as well as elevated blood lactate (Lac; 6.1 mmol/L) and alpha-fetoprotein (AFP; 609.37 ng/ml) levels. Analysis of hemoglobinopathy genes did not reveal any abnormalities. She was treated with antibiotics for infection, blood transfusions to correct anemia, hepatoprotective and choleretic medications to enhance liver function, promote regeneration of liver cells, and mitigate potential damage. On the 11th day of hospitalization (72 days after birth), she developed abdominal distension and vomited stomach contents. The presence of multiple abdominal cavity ascites was confirmed by ultrasound and X-ray. The patient underwent two rounds of abdominal paracentesis to drain the ascites. Routine biochemical tests revealed suppressed albumin levels. However, there were no other abnormalities. After treatment with albumin supplementation, there were slight improvements in the patient’s abdominal distension and pale complexion, but liver functions worsened. The cause of ascite accumulation was not fully established.

**Table 1:** Comparison of coagulation function results from three hospitalizations and outpatient visits

Hospitalization date (Child’s age in days)	PT (s)	APTT (s)	FIB (g/L)	TT (s)	INR
**1st hospitalization (outside hospital)**					
15 d	18.1	101.5	1.3	–	–
**2nd hospitalization (outside hospital)**					
60 d	18.2	68.3	1.36	–	1.14
**3rd hospitalization (our hospital)**					
92 d	18.1	58.1	1.51	6.9	1.53
97 d	17.8	45.3	0.96	9.3	1.51
104 d	15.6	55.7	1.62	9.2	1.32
**Post-discharge outpatient review**					
134 d✮	**15.6**	**55.7**	**1.62**	**10.2**	**1.13**
Reference range	9–15	23–40	2–5	9–15	0.8–1.4

✮: After lactose-restricted formula feeding;-; Missing data; PT: Prothrombin time; APTT: Activated partial thromboplastin time; FIB: Fibrinogen; TT: Thrombin time; INR: international normalized ratio

At 92 days of age, the patient was admitted into the Department of Pediatrics at The First Affiliated Hospital of Guangxi Medical University, China due to jaundice for over 2 months and abdominal distension for 20 days and further diagnosis and treatment of “cholestasis.” She had poor physical growth and development and could lift her head but was unsteady. She had a history of blood transfusions with no adverse reactions and had received hepatitis B and BCG vaccines. Her feeding regimen included a mixture of lactose-free formula and cow’s milk. The patient’s family denied any history of hereditary diseases. There are two healthy older brothers with no similar medical history. The family denied any history of infectious disease exposure.

### Physical examination

General conditions: T:36.6 °C, P:130 beats/min, R:60 breaths/min, weight 3.2 kg (<-3SD), height 55 cm (<-3SD), and age = 92 days. The patient had altered consciousness, jaundice, soft neck, regular breathing, and regular heart rhythms. Her abdomen was distended, veins were visible in the abdominal wall, the liver was subcostal flat umbilical, moderately textured, with blunt margins, the spleen was not palpated under the ribs, the shifting dullness was not cooperative on examination, and bowel sounds were normal. There was mild pitting edema in both lower limbs. Muscle tone was normal in all four limbs, while muscle strength could not be assessed due to poor cooperation. Physiological reflexes were present, while pathological reflexes were not elicited.

### Laboratory examinations

Blood biochemical tests: liver and coagulation functions were abnormal (Table 2). Blood ammonia levels were 85.00 umol/L↑, blood lactate was 3.12 mmol/L↑, AFP was 195.75ng/ml↑, and D-dimer was 1137 ng/ml↑. Ceruloplasmin, blood copper, and G-6-PD were normal. Blood type is B positive for Rh(D). Urine organic acid tests did not reveal any abnormalities. Blood tandem mass spectrometry test revealed decreased free carnitine and various acylcarnitines (free carnitine 4.57 μm↓, acetylcarnitine 4.475 μm↓, propionyl carnitine 0.117 μm↓, butyryl carnitine 0.039 μm↓, isovaleryl carnitine 0.026 μm↓). No abnormalities were found in other biochemical, microbiological, or immunological tests.

**Table 2:** Comparisons of liver function results from three hospitalizations and outpatient visits

Hospitalization date (Child’s age in days)	TBIL (µmol/L)	DBIL (µmol/L)	IBIL (µmol/L)	ALT (U/L)	AST (U/L)	TBA (µmol/L)	ALB (g/L)
**1st hospitalization (outside hospital)**							
8 d	268.4	43.2	225.2	53.2	52.2	146.0	36.5
15 d	87.2	60.6	26.6	41.5	64.3	171.0	35.0
**Outpatient review**							
23 d	80.2	44.4	35.8	149.3	86.4	–	34.4
46 d	102.0	57.4	44.6	39.2	119.4	264.4	33.5
**2nd hospitalization (outside hospital)**							
60 d	63.3	61.7	2.2	40.6	107.2	195.0	32.0
72 d	42.9	33.8	9.1	53.2	90.8	92.0	29.2
80 d	37.2	20.9	13.3	28.9	60.6	51.4	44.2
86 d	62.2	30.0	32.2	130.0	290.8	–	–
**3rd hospitalization (our hospital)**							
92 d	47.0	35.9	11.1	46.0	76.0	299.1	36.1
97 d	68.9	46.8	22.1	28.0	83.0	186.4	34.5
104 d	64.7	48.9	15.8	100.0	51.0	133.5	29.4
**Post-discharge outpatient review**							
**134 d**✮	**6.0**	**2.8**	**3.2**	**34.0**	**56.0**	**11.0**	**40.0**
Reference range	3.4–20.5	0–6.8	3.1–14.3	7–45	13–40	0–10	40–55

✮: After lactose-restricted formula feeding;-; Missing data; TBIL: Total bilirubin; DBIL: Direct bilirubin; IBIL: Indirect bilirubin; ALT: Alanine aminotransferase; AST: Aspartate aminotransferase; TBA: Total bile acids; ALB: Albumin

### Imaging examinations

Abdominal ultrasound: enlarged liver with uneven surface and a high echogenic mass in porta hepatis, implying reactive lymph nodes; atrophic and distorted gallbladder and ascites (approximately 5.5 cm deep). Magnetic resonance cholangiopancreatography (MRCP) did not reveal any abnormalities.

### Preliminary diagnosis and treatment

Preliminary diagnosis: (i) Infantile hepatitis syndrome, (ii) Ascites (cause to be investigated), (iii) Moderate anemia, (iv) Metabolic acidosis, (v) Hyperlactatemia, (vi) Coagulation dysfunction, and (vii) Malnutrition. Treatment included feeding the infant lactose-restricted formula, extensively hydrolyzed formula, protecting and promoting liver and gallbladder functions, correcting anemia, and supplementing coagulation factors and carnitine. After the above treatments, the liver and coagulation functions improved to some extent.

A possible inherited metabolic disorder was suspected. Informed consent form for blood collection was signed by the guardian, and sample was sent to Wuhan Kangshengda Medical Laboratory for whole-exome sequencing.

### Genetic test results

**Fig. 2:**
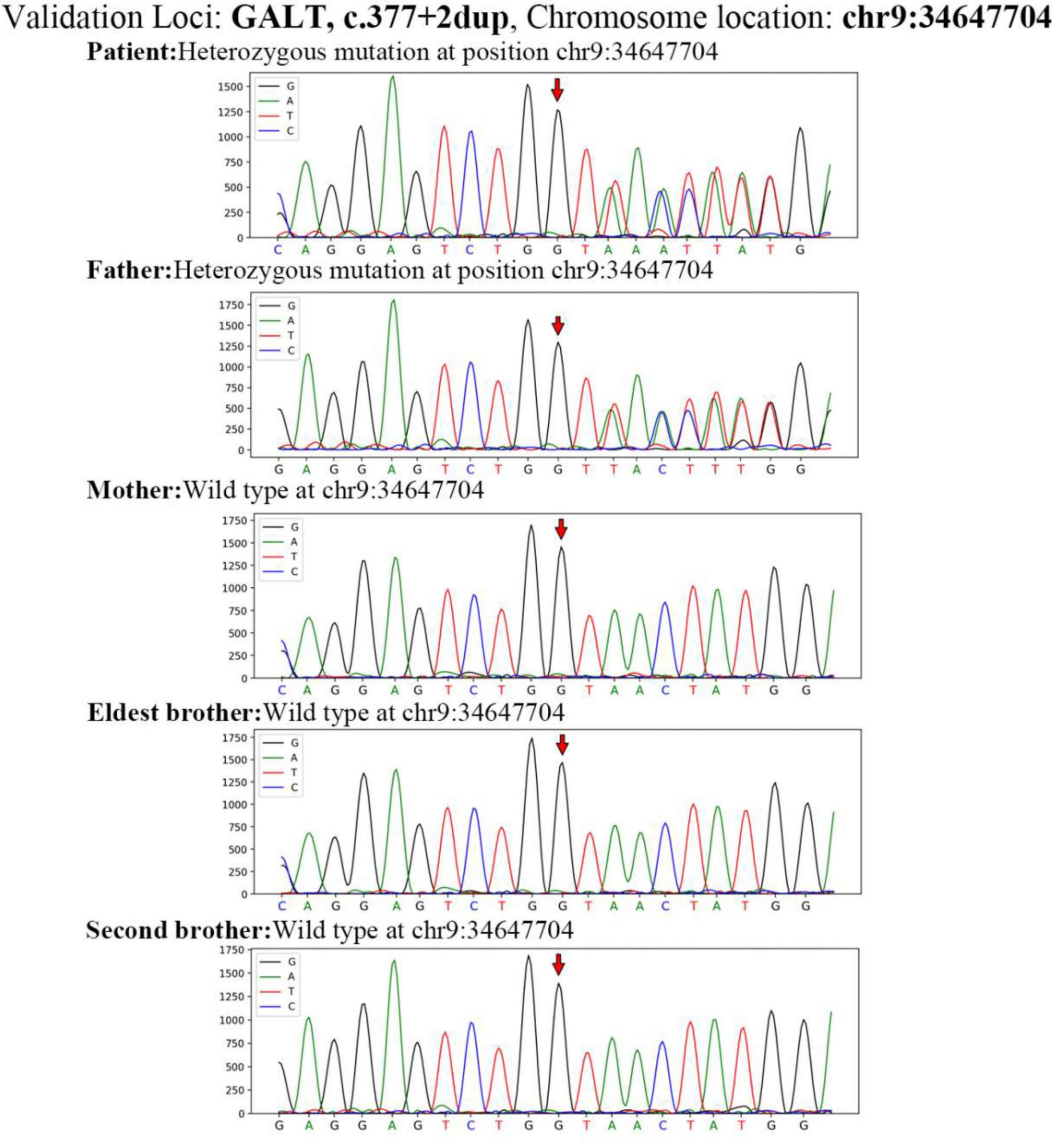
Sanger sequencing confirmed that the patient was a compound heterozygote for the c.377 + 2dup variant of the *GALT* gene located at chromosome position chr9:34647704. The father was also a compound heterozygote with the same variant, whereas the mother and elder brothers were wild types

**Fig. 3:**
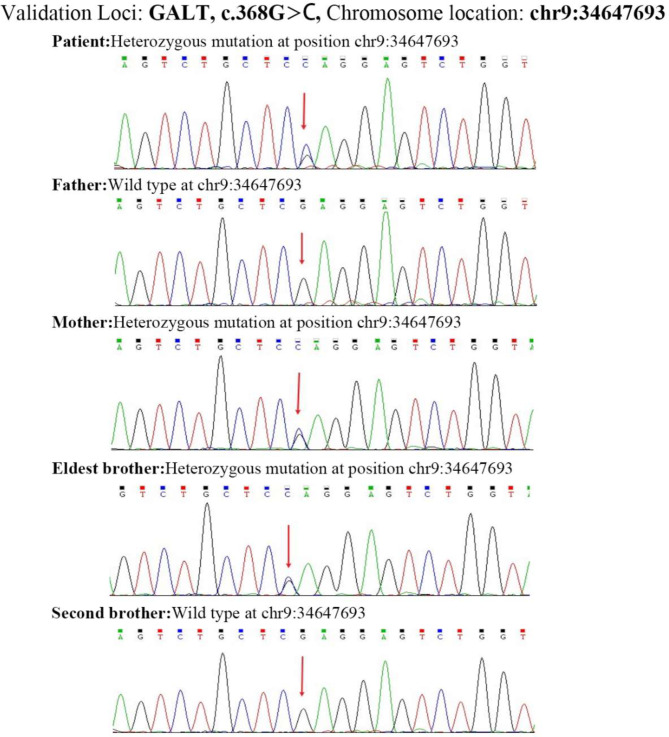
Sanger sequencing confirmed that the patient was heterozygous for the c.368G > C variant in *GALT* (chromosome position: chr9:34647693), with the father being wild type and the mother and eldest brother being heterozygous for the same variant, while the second brother is wild type

Whole-exome sequencing revealed two potentially pathogenic heterozygous variants in the *GALT* gene c.377 + 2dup (located in intron 5) and c.368G > C (p.Arg123Pro; located in exon 4) of chromosome 9 and transcript NM_000155. The c.377 + 2dup variant is a classic splice site variant that cannot predict the amino acid change it causes. While c.377 + 2dup has been reported in earlier studies, it was not widely reported in the Human Gene Mutation Database (HGMD) before this investigation. The c.368G > C (p.Arg123Pro) is a missense variant. A pathogenic variant (c.368G > A (p.R123Q)) at the same site has been reported in the HGMD database; however, it results in a different amino acid change compared to the variant in this case [14]. It has been postulated that this variant may be pathogenic; however, it has yet to be identified in the East Asian population or reported in the gnomAD database. According to the standards and guidelines of the American College of Medical Genetics and Genomics (ACMG) [15], the first variant meets the criteria PM2, while the second variant meets criteria PM1, PM2, and PM3. Although PVS1 does not apply to the c.377 + 2dup variant since it does not map to the canonical donor site, PM3 could be considered, especially considering the homozygous patient already described; however, even with this consideration, the variant would remain categorized as a Variant of Uncertain Significance (VOUS). This study revealed that the c.368G > C variant meets the PM5 criterion, as it involves a different amino acid change known to be associated with pathogenic or likely pathogenic variants. Additionally, computational prediction tools, including REVEL, consistently predict a deleterious impact on the protein, satisfying the PP3 criterion. The variants were verified by Sanger sequencing and found to be inherited from the parents, who were carriers of heterozygous variants (Figs. 2 and 3). This is consistent with an autosomal recessive inheritance pattern. After considering the patient’s clinical features and treatment characteristics, a diagnosis of classical galactosemia was made.

## Discussion

Based on biochemical phenotypes, genotypes, and potential development of acute and long-term complications, classical galactosemia (type I galactosemia), characterized by *GALT* enzyme deficiencies, can be classified into three primary forms: (1) Classical galactosemia; (2) Clinical variant galactosemia, and (3) Biochemical variant galactosemia [16].

According to HGMD, there are currently 319 known variants in the *GALT* gene. These variants can be classified into several types, including 251 missense and nonsense variants, 27 splice site variants, 24 small deletion variants, 5 insertion variants, 3 insertion-deletion variants, 8 large deletion variants, and 1 complex rearrangement [17]. It has been reported that *GALT* is mainly affected by missense variants, followed by splice site variants, which is consistent with our genetic test results (Table 3). The mechanisms by which missense variants lead to the reduction or loss of *GALT* enzyme activity have yet to be fully established. Current research suggests that misfolding of the enzyme may be the fundamental cause of the decrease in activity [18]. There are two main theories: one suggests that the gene variant directly causes a reduction or loss of enzyme activity; the other proposes that the variant forms an unstable polypeptide chain (protein) [19].

**Table 3:** Results of *GALT* gene variants in 12 cases of classical Galactosemia in China (from 2017 to 2022)

Case	Gene	Nucleotide change	Amino acid change	Variant type	Zygotic type	Origin	Site of variant (exon no.)
Case1 [19]	*GALT*	c.377 + 2dupT	/	Splice site	homozygous	Parents	Exon4
Case2 [19]	*GALT*	c.558 C > G	p.H186Q	Missense	heterozygous	Father	Exon6
	*GALT*	c.958G > C	p.A320T	Missense	heterozygous	Mother	Exon10
Case3 [19]	*GALT*	c.691 C > G	p.R231G	Missense	heterozygous	Father	Exon8
	*GALT*	c.983G > A	p.R328H	Missense	heterozygous	Mother	Exon10
Case4 [19]	*GALT*	c.367 C > T	p.R123X	Missense	homozygous	Parents	Exon4
Case5 [13]	*GALT*	c.904 + 1G > T	/	Splice site	heterozygous	Father	–
	*GALT*	c.687G > A	p.K229K	Samesen-se	heterozygous	Mother	–
Case6 [10]	*GALT*	c.378–2 A > T	/	Splice site	heterozygous	Father	Exon5
	*GALT*	c.1018G > A	p.E340K	Missense	heterozygous	Mother	Exon10
Case7 [20]	*GALT*	c.564G > C	p.Q188H	Missense	heterozygous	Father	–
	*GALT*	c.116 A > T	p.D39V	Missense	heterozygous	Mother	–
Case8 [20]	*GALT*	c.754 C > T	p.Q252H	nonsense	heterozygous	Father	–
	*GALT*	c.904 + 1G > T	/	Splice site	heterozygous	Mother	–
Case9 [21]	*GALT*	c.982 C > T	p.R328C	Missense	heterozygous	Father	–
	*GALT*	c.1064 C > T	p.A355V	Missense	heterozygous	Mother	–
Case10 [22]	*GALT*	c.829T > C	p.S277Pro	Missense	heterozygous	Father	Exon9
	*GALT*	c.970 C > G	p.P324A	Missense	heterozygous	Mother	Exon10
Case11 [23]	*GALT*	c.396 C > G	p.H132Q	Missense	heterozygous	Mother	–
	*GALT*	c.974 C > T	p.P325L	Missense	heterozygous	Father	–
Case12 [23]	*GALT*	c.974 C > T	p.P325L	Missense	homozygous	Parents	–
Reported Case	*GALT*	c.377 + 2dup	/	Splice site	heterozygous	Father	Exon9
	*GALT*	c.368G > C	p.Arg123Pro	Missense	heterozygous	Mother	Exon9

*Note* - : Not recorded; / : Not predictable

*GALT* gene variants exhibit significant ethnic and regional differences; c.563 A > G (p.Gln188Arg) is common in European populations. It is the most frequently reported variant in literature, accounting for about 70% of all mutant alleles. The c.855G > T (p.Lys285Asn) variant is common in German and Austrian populations, accounting for about 54% of all variant alleles. In contrast, the c.404 C > T (p.Ser135Leu) variant is common in African American populations, accounting for about 50% of all variant alleles. The c.253–2 A > G variant is common in Hispanic populations, accounting for about 11% of all variant alleles, while large deletions of 5 kb and 5.5 kb are common in Ashkenazi Jewish populations. In the Japanese population, the common variants include c.[940 A > G; c.-116-119delGTCA] (p.Asn314Asp) and p.R231H [3, 5, 24, 25]. There are no reports of a predominant type of *GALT* gene variant in the Chinese population. Searching the Chinese database (CNKI) and PubMed, we found 12 cases of *GALT* variants reported in China in the past 5 years, and their variant types differ from the above-reported common variants (Table 3). Differences in variants among different populations are the fundamental reason for the diversity of its clinical manifestations. Compound heterozygous variants of c.377 + 2dup and c.368G> C (p.Arg123Pro) in the *GALT* gene, which we reported, are variant sites and will further expand the spectrum of *GALT* gene variants. In addition to its association with classic galactosemia, the c.377 + 2dup variant has been previously reported in a cohort of patients with intrahepatic cholestasis [26]. Further investigations are warranted to elucidate any potential implications and associations related to this variant in the context of intrahepatic cholestasis.

Classical galactosemia is associated with genotypes resulting in severe *GALT* deficiencies, such as Q188R/Q188R, K285N/K285N, L195P/L195P, and those comprising two different variants, e.g., Q188R and K285N. These genotypes have little to no detectable enzyme activity in red blood cells and the liver [27]. Biochemical variant galactosemia (Duarte galactosemia) is commonly caused by one galactosemia allele (e.g., Q188R, K285N, etc.) and one Duarte allele. This gene is associated with a promoter deletion c.-119_-116delGTCA (Deficiency of UDPglucose-hexose-1-phosphate uridylyltransferase) and missense variant N314D. There are three intronic variants in the cis-configuration (c.378-27G > C, c.507 + 62G > A, and c.508-24G > A) [28]. Podskarbi et al. [29] reported that substitutions in these three introns may alter mRNA, and in vitro, the 5’UTR deletion reduced *GALT* gene transcription by about 55% [30]. Despite this, it is worth noting that *GALT* enzyme activity can range from 40 to 50% [31, 32], and individuals with this enzyme activity typically do not experience clinical symptoms [3].

Most countries, including China, do not routinely conduct newborn screening (NBS) for galactosemia, essential for early disease identification. Some developed Western countries, including Japan (100%) [33], Lithuania (100%) [12], approximately one-third of European countries (39.2%) [16], and the United States (100%) [26], have implemented routine NBS. Quantitative measurements of *GALT* activity in red blood cells are the gold standard for diagnosing this disease, and this method can also identify variants with partial enzyme activity [34, 35]. Welling et al. postulated that if the detected variants in the GALT gene are reported as pathogenic, and present in compound heterozygous state, it is enough to confirm the diagnosis of Galatosemia [3, 36]. Genetic analysis in the present case identified one likely pathogenic variant and one variant of uncertain significance. As identified variants are in trans configuration, and the patient’s clinical symptoms are consistent with the diagnosis, Galactosemia can be confirmed in the patient.

The patient, in this case, developed jaundice of unknown cause three days after birth, followed by hepatomegaly and abnormal liver functions. After three hospitalizations and a series of examinations, infection-related and non-infection-related factors, such as Hepatolenticular degeneration (HLD) and Niemann-Pick disease (NPD), were ruled out. Therefore, non-infectious diseases commonly associated with liver dysfunctions, such as galactosemia, were prioritized. Only through genetic testing were we able to confirm the correlation between the infant’s abnormal liver and coagulation functions with galactosemia.

According to the international clinical treatment guidelines for classical Galactosemia (GalNet) [3] and experts such as Welling, when a child is suspected of having classical galactosemia, the clinician should immediately begin restricting galactose intake (such as avoiding legumes, casein hydrolysates, or elemental formula) rather than waiting for a confirmed diagnosis. For those with a confirmed diagnosis, lactose intake should be restricted for their entire lives. Lactose intake should be excluded for children with red blood cell *GALT* enzyme activity < 10% and/or pathological variations in both *GALT* alleles (including p.S135L). Evidence to determine whether treatment is necessary for children with residual *GALT* activity of 10%∼15% is insufficient. Children with Duarte variations are not recommended for treatment.

## Conclusions

A galactose-restricted diet is effective for treating neonatal complications but is not sufficient for treating long-term complications. From positive results with animal models, there is reason to hope that gene and mRNA therapy may restore human GALT activity in the future, but currently early detection followed by a lactose-restricted diet is the most effective treatment for classic galactosemia. This child was not diagnosed until three months of age. All clinical features of the affected child were consistent with classic galactosemia. The disease was ultimately diagnosed via genetic testing. After diagnosis, the child was fed with a lactose-restricted formula, and jaundice subsided significantly while liver functions improved. Further follow-up was conducted. Unfortunately, we did not recognize and restrict lactose in the early stages. By reporting the diagnosis and treatment processes of this patient, we aim to draw the attention of clinicians and encourage early recognition and intervention, which can effectively reduce or prevent the occurrence of more severe complications. We suggest that newborns suspected of the disease undergo NBS and genetic testing as early as possible to avoid misdiagnosis.

## Data Availability

The datasets generated and/or analyzed during the current study are available in the Genome Sequence Archive (GSA) repository: https://ngdc.cncb.ac.cn/gsa-human/s/rnxNzs70.

## Abbreviations

*GALT*: Galactose-1-phosphate uridylyltransferase
GALK: Galactokinase
GALM: Galactose epimerase
GALE: UDP-galactose-4´-epimerase
HGMD: Human Gene Mutation Database
GnomAD: Genome aggregation database
